# Effect of a robotic insole-type active assist device on horizontal ground reaction force and center-of-pressure stability during stepping in patients with medial knee osteoarthritis

**DOI:** 10.3389/frobt.2026.1898805

**Published:** 2026-08-12

**Authors:** Taku Itami, Takaaki Aoki, Haruhiko Akiyama

**Affiliations:** 1 Department of Rehabilitation and Orthopedics, Gifu University, Gifu, Japan; 2 Department of Electronics and Bioinformatics, School of Science and Technology, Meiji University, Kawasaki, Kanagawa, Japan

**Keywords:** ankle alignment correction, center of pressure, ground reaction force, insole-type active assist device, knee osteoarthritis, robotic insole-type device, stepping

## Abstract

An insole-type active assist device has been developed as a robotic system to dynamically correct ankle alignment at heel contact in patients with medial knee osteoarthritis. Although our previous feasibility study demonstrated that the device could be safely used during an on-the-spot stepping task, its effects on loading behavior remain unclear. This study aimed to investigate whether dynamic ankle alignment correction using the device alters horizontal ground reaction force variability and center-of-pressure sway during stepping. Force-plate data obtained from six ambulatory patients with medial knee osteoarthritis were analyzed as a secondary biomechanical analysis. Each participant performed repeated stepping trials under two conditions: a non-control condition, in which the device was worn without motor control, and a control condition, in which heel-eversion assistance was provided. Stance phases were extracted using vertical ground reaction force, and horizontal ground reaction force components, center-of-pressure sway measures, peak vertical ground reaction force, and vertical impulse were calculated. Compared with the non-control condition, the control condition reduced the ranges of anterior–posterior and medial–lateral ground reaction force components in all participants. Center-of-pressure rectangular sway area also decreased consistently, and trajectory length tended to decrease, whereas peak vertical ground reaction force and vertical impulse showed little change. These findings suggest that dynamic ankle alignment correction using a robotic insole-type device may reduce horizontal loading variability and center-of-pressure sway during stepping without substantially altering vertical loading. Because this study was based on a small sample and an exploratory secondary analysis, the findings should be interpreted as preliminary biomechanical evidence rather than evidence of clinical effectiveness.

## 1 Introduction

Knee osteoarthritis (OA) is a highly prevalent musculoskeletal disorder among older adults and is a major cause of pain, mobility limitation, and reduced quality of life (Murphy et al., 2008). Medial knee OA is frequently associated with varus lower-limb alignment and increased mechanical loading of the medial tibiofemoral compartment. During weight-bearing activities, excessive or poorly controlled loading of the lower limb can contribute to pain, dynamic instability, and functional decline. Therefore, biomechanical interventions that can modify lower-limb loading during functional movements are important for patients with medial knee OA.

Dynamic frontal-plane instability is one of the key biomechanical concerns in medial knee OA. Varus thrust, which is characterized by an abrupt worsening of varus alignment during the stance phase of gait, has been associated with the progression of knee OA (Chang et al., 2004). Quantitative assessments using motion analysis have also shown that varus thrust is frequently observed in patients with medial knee OA and is related to static and dynamic alignment parameters (Kuroyanagi et al., 2012). These findings suggest that interventions targeting dynamic lower-limb loading and frontal-plane instability may be clinically relevant in this population. Recent studies have reported that the progression of knee OA is influenced not only by lower-limb alignment and external knee joint moments but also by disruption of intra-articular load distribution associated with meniscal root tears and meniscal extrusion (Foreman et al., 2021; Canton et al., 2026). In addition, conservative biomechanical interventions for medial knee OA, including lower-limb orthoses and foot- or ankle-mediated gait modifications, have been investigated to modify knee loading, alignment, pain, and function (Mahmoodi et al., 2024; Uhlrich et al., 2025). These findings support the importance of interventions that modify load transfer and lower-limb alignment in patients with knee OA. However, most conventional approaches provide static correction or task-level gait modification, and dynamic correction of ankle alignment specifically at heel contact remains insufficiently explored.

The mechanical loading of the knee is influenced not only by knee alignment but also by the interaction among the foot, ankle, shank, and ground during the stance phase. In particular, foot and ankle alignment can affect the ground reaction force vector, center-of-pressure trajectory, and load-bearing axis of the lower limb. Among conservative biomechanical interventions, lateral wedge insoles have been widely investigated for medial knee OA. By modifying the inclination of the foot–ground interface, lateral wedge insoles can alter foot–ground interaction, center-of-pressure behavior, and lower-limb biomechanics (Hinman et al., 2009; Du et al., 2024). Previous studies have reported that lateral wedge insoles may reduce knee adduction moment or induce biomechanical changes in some patients with knee OA (Hinman et al., 2009; Ferreira et al., 2022; Du et al., 2024). However, their effects are not always consistent, partly because conventional insoles provide static correction throughout the stance phase and cannot adapt to step-to-step variations in ankle posture, foot placement, or the timing of dynamic malalignment.

To address this limitation, we developed an insole-type active assist device that dynamically corrects ankle alignment at heel contact. The device is designed to provide heel-eversion assistance at heel contact, thereby guiding an inverted ankle position toward a more neutral alignment during stepping. In our previous feasibility study, we demonstrated that the device could be safely used during an on-the-spot stepping task in patients with medial knee OA and that motion-capture-based evaluation could be successfully conducted (Itami et al., 2026). However, that study was primarily designed to examine feasibility, including safety, protocol completion, device operability, and data acquisition. It was not intended to determine the biomechanical effectiveness of the device or to clarify how dynamic ankle alignment correction influences ground reaction force and center-of-pressure behavior.

Force-plate analysis provides useful information regarding the mechanical interaction between the lower limb and the ground during stepping and walking. In addition to vertical ground reaction force, horizontal ground reaction force components and center-of-pressure displacement provide important information about loading stability during the stance phase. Center-of-pressure-based measures have been widely used to evaluate postural steadiness and stability (Winter, 1995; Prieto et al., 1996). In the context of medial knee OA, excessive horizontal force variability or increased center-of-pressure sway during stepping may reflect unstable weight acceptance or insufficient control of lower-limb loading. If dynamic ankle alignment correction stabilizes lower-limb loading, it may reduce horizontal ground reaction force variability and center-of-pressure sway without necessarily changing the magnitude of vertical loading.

Therefore, the purpose of this study was to investigate the effect of an insole-type active assist device on horizontal ground reaction force and center-of-pressure stability during on-the-spot stepping in patients with medial knee OA. This study was conducted as a secondary force-plate analysis of data obtained during repeated stepping trials under non-control and control conditions. We hypothesized that the control condition would reduce horizontal ground reaction force variability and center-of-pressure sway, while vertical ground reaction force peak and vertical impulse would remain largely unchanged.

## 2 Materials and methods

### 2.1 Study design

This study was conducted as a secondary force-plate analysis of data obtained in a previously reported feasibility study of an insole-type active assist device for ankle alignment correction during stepping in patients with medial knee osteoarthritis (Itami et al., 2026). The previous study primarily evaluated the feasibility and safety of the device, including participant recruitment, protocol completion, device operability, and motion-capture-based measurement. In contrast, the present study focused on the biomechanical effects of the device on ground reaction force and center-of-pressure behavior during stepping.

Each participant performed repeated on-the-spot stepping trials under two conditions: a non-control condition, in which the device was worn but motor control was disabled, and a control condition, in which heel-eversion assistance was provided by the device. In the present analysis, the non-control condition was treated as the reference condition, and the control condition was evaluated to determine whether dynamic ankle alignment correction altered horizontal ground reaction force variability and center-of-pressure sway.

### 2.2 Participants

Six ambulatory patients with medial knee osteoarthritis were included in this secondary analysis. All participants had been diagnosed with medial knee osteoarthritis by an orthopedic physician and were classified as Kellgren–Lawrence grade II or III. The cohort consisted of two males and four females, with a mean age of 67.8 ± 7.4 years. Four participants were classified as Kellgren–Lawrence grade II and two as grade III. The mean femoro–tibial angle was 187.5° (range, 184°–193°), indicating mild varus alignment. The mean body mass index was 25.5 ± 1.7 kg/m^2^. The mean knee pain score assessed using the visual analog scale was 49.2 ± 9.1 mm, and the mean foot posture index was −3.0 ± 0.8, indicating a slightly supinated foot posture. Participants were able to perform the on-the-spot stepping task without external assistance. Individuals who had difficulty performing the task or were judged by a physician to be unsuitable for participation were excluded. All participants were confirmed by an orthopedic physician to have no severe bilateral deformity or significant difference in knee range of motion between the left and right knees.

All participants provided written informed consent before participation. The experimental procedures were approved by the Ethics Review Committee for Studies Involving Human Subjects at Gifu University School of Medicine, Japan, approval number 2023–197, and were conducted in accordance with the Declaration of Helsinki.

### 2.3 Insole-type active assist device

The insole-type active assist device used in this study was designed to dynamically correct ankle alignment at heel contact. The device has a compact in-shoe structure with a heel-tilting mechanism that rotates the heel section in the frontal plane toward eversion. The device is intended to guide an inverted heel position toward a neutral, approximately horizontal alignment at the time of heel contact. From a biomechanical perspective, abnormal ankle posture at heel contact may alter lower-limb loading mechanics during stance, and excessive ankle inversion has been considered to shift the ground reaction force vector medially relative to the knee joint center, thereby contributing to increased knee adduction moment and varus thrust in patients with medial knee OA (Neumann, 2010; Chang et al., 2004; Kuroyanagi et al., 2012). Guiding the heel toward a neutral or slightly everted position is therefore expected to laterally shift the center-of-pressure and realign the load-bearing axis of the lower limb, which is consistent with the biomechanical rationale of lateral wedge insoles (Yılmaz et al., 2016; Ferreira et al., 2022).

**FIGURE 1:**
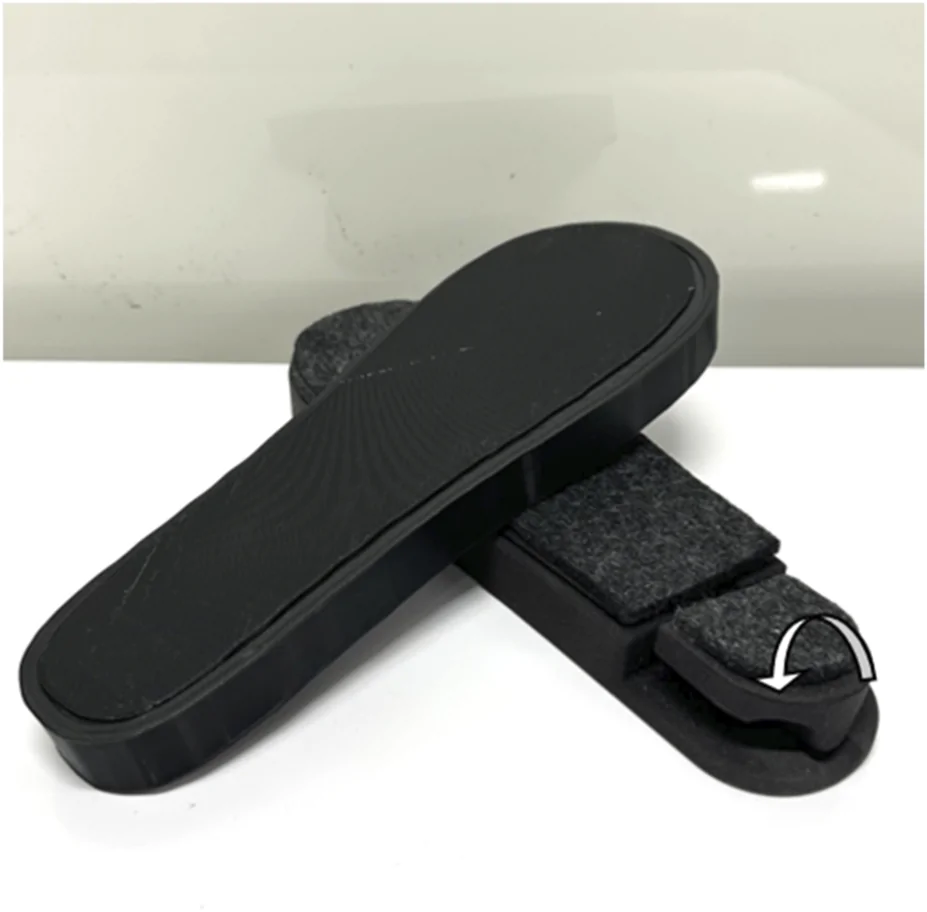
Overview of the robotic insole-type active assist device. The device was designed to dynamically correct ankle alignment at heel contact using a heel-tilting mechanism. After switch activation, the heel section provides eversion assistance to guide the ankle toward a neutral or slightly everted alignment.

The prototype consisted of an Arduino Nano microcontroller, a six-axis inertial sensor (MPU-6050, InvenSense Co., San Jose, CA, United States), and a stepping motor (PKP213D05A, Oriental Motor Co., Tokyo, Japan). The device weighed approximately 500 g and had a thickness of 22 mm. The device size was 23.5 cm for female participants and 27 cm for male participants. As a device-related durability specification, the device was previously reported to endure a 75 kg × 1.2 load applied 20 mm from the center axis of the heel for 300 cycles. The mechanical correction angle ranged from 0° to 10°, corresponding to eversion correction from an inverted heel position toward neutral alignment. The mechanism was capable of rotating from 0° to 10° within 500 ms. An overview of the robotic insole-type active assist device is shown in Figure 1. Detailed specifications and feasibility outcomes of the device have been reported previously (Itami et al., 2026).

In the experimental setup, the active device was attached to the right foot, while a dummy device with the same shape and weight was attached to the left foot. All participants wore standardized shoes during the experiment to reduce footwear-related variability. Accordingly, the right limb was analyzed in all participants. The analyzed limb was standardized as the right side for the experimental protocol and did not necessarily correspond to the most symptomatic knee in every participant.

### 2.4 Experimental procedure

Participants performed repeated on-the-spot stepping trials under two conditions: non-control and control. In the non-control condition, the participants wore the device, but the heel-eversion control mechanism was disabled. In the control condition, the device provided heel-eversion assistance during stepping. A physician manually activated the device switch approximately 500 ms before heel contact during each stepping motion. In the present prototype protocol, excessive ankle inversion was not automatically detected using a predefined sensor threshold; instead, device activation was determined by the physician-operated switch. Before data collection, the physician performed sufficient practice to standardize the switching procedure and to ensure that the activation timing corresponded to the expected heel-contact phase. After switch activation, the heel section rotated toward eversion within the mechanical correction range of 0°–10°, so that the heel was intended to reach a neutral, approximately horizontal alignment at the time of heel contact. Therefore, the magnitude of correction was mechanically limited by the device, and the timing of correction was determined by the physician-operated switch in this prototype protocol.

Each condition consisted of repeated stepping trials. Participants were instructed to step in place at a comfortable rhythm while maintaining a stable posture. Cadence and step timing were not externally controlled using a metronome or other pacing device. Stance time was measured from the force-plate data based on the vertical ground reaction force threshold and was included as a descriptive variable. The stepping task was selected instead of continuous walking to ensure participant safety and to accommodate the wired prototype configuration. Sufficient practice was provided before data collection. The order of experimental procedures and the general testing environment were the same as those described in the previous feasibility study (Itami et al., 2026).

### 2.5 Force-plate data acquisition

Ground reaction force and center-of-pressure data were recorded using force plates (AMTI, Watertown, MA, United States) integrated with a Vicon motion-capture system during the stepping task. The force-plate data included three-dimensional ground reaction force components, three-dimensional moment components, and center-of-pressure coordinates. The analog force-plate signals were sampled at 1,000 Hz.

The coordinate system used in the present analysis was based on the exported force-plate data. The vertical ground reaction force component was used to identify the stance phase of each step. The horizontal ground reaction force components were analyzed to evaluate loading variability in the anterior–posterior and medial–lateral directions. Center-of-pressure displacement was used to evaluate sway behavior during the stance phase.

### 2.6 Data processing

Force-plate data were processed using custom analysis scripts. The stance phase of each step was identified using the vertical ground reaction force. A threshold of 50 N was applied to detect foot contact. Periods in which the vertical ground reaction force exceeded this threshold were defined as stance phases. Incomplete stance phases at the beginning or end of each recording were excluded from the analysis.

For each valid stance phase, ground reaction force and center-of-pressure variables were calculated. The analyzed steps were then averaged within each participant and condition. The participant-level mean values were used for group-level comparison between the non-control and control conditions.

### 2.7 Outcome measures

The primary outcomes were horizontal ground reaction force variability and center-of-pressure sway measures during the stance phase. Horizontal ground reaction force variability was evaluated using the ranges of the anterior–posterior and medial–lateral ground reaction force components. The absolute peak values of the horizontal ground reaction force components were also calculated for each valid stance phase. The range-based measures were selected because the present study focused on the within-stance excursion of horizontal loading during each stepping movement. The anterior–posterior and medial–lateral ground reaction force ranges represent the difference between the maximum and minimum horizontal force values during a stance phase and therefore provide a simple index of the magnitude of horizontal loading fluctuation within each step. Although range-based measures may be sensitive to extreme values, incomplete stance phases were excluded, and step-level values were averaged within each participant and condition to reduce the influence of isolated fluctuations. Other variability measures, such as standard deviation, coefficient of variation, impulse, or step-to-step variability, may provide complementary information but were not selected as primary outcomes because they do not directly represent the within-stance excursion of horizontal ground reaction force targeted in the present analysis.

Center-of-pressure behavior was evaluated using the range of center-of-pressure displacement in the anterior–posterior and medial–lateral directions, center-of-pressure trajectory length, and center-of-pressure rectangular sway area. The center-of-pressure trajectory length was calculated as the cumulative distance traveled by the center of pressure during the stance phase:

$${COP}_{length}=\sum_{i=1}^{n-1}\sqrt{{\left(COP_{AP,i+1}-COP_{AP,i}\right)}^{2}+{\left(COP_{ML,i+1}-COP_{ML,i}\right)}^{2}}$$

where ${COP}_{length}$ is the center-of-pressure trajectory length; $COP_{AP,i}$ and $COP_{ML,i}$ are the anterior–posterior and medial–lateral center-of-pressure coordinates at the i -th sample, respectively; and n is the number of samples during the stance phase.

The center-of-pressure rectangular sway area was calculated as the product of the anterior–posterior and medial–lateral center-of-pressure ranges:

$$COP_{area}=COP_{AP,range}\times COP_{ML,range}$$

where $COP_{area}$ is the center-of-pressure rectangular sway area, and $COP_{AP,range}$ and $COP_{ML,range}$ are the ranges of center-of-pressure displacement in the anterior–posterior and medial–lateral directions, respectively.

To confirm whether changes in horizontal force and center-of-pressure behavior were accompanied by changes in overall vertical loading, peak vertical ground reaction force and vertical impulse were calculated as secondary outcomes. Vertical impulse was calculated as the time integral of the vertical ground reaction force over the stance phase using rectangular integration:

$$Impulse_{Z}=\sum_{i=1}^{n}GRF_{Z,i}\Delta t$$

where $Impulse_{Z}$ is the vertical impulse, $GRF_{Z,i}$ is the vertical ground reaction force at the i -th sample, n is the number of samples during the stance phase, and Δt is the sampling interval. Because the force-plate data were sampled at 1,000 Hz, Δt was 0.001 s. All variables were first calculated for each valid stance phase and then averaged within each participant and condition.

### 2.8 Statistical analysis

Because this study was conducted as a secondary exploratory analysis with a small sample size, both descriptive and inferential statistics were used cautiously. For each outcome, participant-level mean values were calculated for the non-control and control conditions. Group-level results were summarized as mean and standard deviation.

The direction of change between conditions was examined for each participant. Paired comparisons between the non-control and control conditions were performed using the Wilcoxon signed-rank test as the primary exploratory statistical test because of the small sample size. In addition, paired t-tests, standardized effect sizes, and 95% confidence intervals for the mean paired differences were calculated as supplementary exploratory indicators to support interpretation of the magnitude and uncertainty of change. The paired difference was calculated as the control condition minus the non-control condition. Statistical significance was set at p < 0.05. No formal correction for multiple comparisons was applied because the present study was an exploratory secondary analysis intended to generate preliminary biomechanical evidence rather than to confirm definitive treatment effects. Therefore, p-values were interpreted cautiously and were considered together with the consistency of change across participants, effect sizes, confidence intervals, and biomechanical relevance.

## 3 Results

### 3.1 Analyzed steps

Force-plate data were successfully analyzed for all six participants under both the non-control and control conditions. After excluding incomplete stance phases at the beginning and end of each recording, the number of analyzed steps was 12 and 15 for Participant 1, 12 and 13 for Participant 2, 12 and 11 for Participant 3, 12 and 10 for Participant 4, 11 and 12 for Participant 5, and 11 and 12 for Participant 6 under the non-control and control conditions, respectively. Thus, all participants had sufficient valid stepping trials for within-participant comparison. The number of analyzed steps for each participant is shown in Table 1.

**TABLE 1:** Number of analyzed steps for each participant.

Participant	Non-control condition	Control condition
P1	12	15
P2	12	13
P3	12	11
P4	12	10
P5	11	12
P6	11	12

### 3.2 Vertical ground reaction force

Vertical loading measures showed little difference between the non-control and control conditions. The peak vertical ground reaction force was 564.4 ± 119.5 N in the non-control condition and 560.1 ± 124.6 N in the control condition. The vertical impulse was also similar between conditions, with values of 427.9 ± 137.4 Ns in the non-control condition and 427.8 ± 141.8 Ns in the control condition. These results indicate that the overall magnitude of vertical loading during stepping was largely maintained between conditions.

### 3.3 Horizontal ground reaction force

In contrast to vertical loading, horizontal ground reaction force variability decreased under the control condition. The absolute peak value of the anterior–posterior ground reaction force component decreased from 18.4 ± 5.4 N in the non-control condition to 13.7 ± 3.6 N in the control condition. This decrease was observed in all six participants. The range of the anterior–posterior ground reaction force component also decreased from 27.0 ± 6.6 N to 21.4 ± 4.1 N, and this reduction was again observed in all participants.

The medial–lateral ground reaction force component showed a similar tendency. The absolute peak value of the medial–lateral component decreased from 47.5 ± 16.2 N in the non-control condition to 43.9 ± 14.1 N in the control condition. The range of the medial–lateral ground reaction force component decreased from 57.4 ± 12.5 N to 52.7 ± 13.8 N. The reduction in medial–lateral ground reaction force range was observed in all six participants. These findings suggest that the control condition reduced horizontal loading variability during the stance phase.

### 3.4 Center-of-pressure behavior

Center-of-pressure measures also showed reduced sway under the control condition. The anterior–posterior center-of-pressure range decreased from 111.4 ± 18.0 mm in the non-control condition to 102.9 ± 18.9 mm in the control condition. The medial–lateral center-of-pressure range decreased from 33.6 ± 11.6 mm to 29.5 ± 11.6 mm.

The center-of-pressure trajectory length decreased from 243.1 ± 63.6 mm in the non-control condition to 211.0 ± 50.4 mm in the control condition. In addition, the center-of-pressure rectangular sway area decreased from 3,793.4 ± 1,485.5 mm^2^ to 3,103.0 ± 1,498.4 mm^2^. The reduction in center-of-pressure sway area was observed in all six participants. These results indicate that the control condition reduced center-of-pressure sway during stepping. A summary of the force-plate variables under the non-control and control conditions is presented in Table 2.

**TABLE 2:** Summary of force-plate variables under the non-control and control conditions. Note: Values are presented as mean ± standard deviation based on participant-level means. Mean difference was calculated as control condition minus non-control condition. Participants showing reduction indicates the number of participants whose value decreased under the control condition compared with the non-control condition. GRF, ground reaction force; COP, center of pressure; AP, anterior–posterior; ML, medial–lateral.

Variable	Non-control condition	Control condition	Mean difference	Participants showing reduction
Stance time (s)	1.08 ± 0.24	1.03 ± 0.17	−0.05	3/6
Peak vertical GRF (N)	564.4 ± 119.5	560.1 ± 124.6	−4.3	3/6
Vertical impulse (Ns)	427.9 ± 137.4	427.8 ± 141.8	−0.1	3/6
Absolute peak AP-GRF (N)	18.4 ± 5.4	13.7 ± 3.6	−4.7	6/6
Absolute peak ML-GRF (N)	47.5 ± 16.2	43.9 ± 14.1	−3.7	4/6
AP-GRF range (N)	27.0 ± 6.6	21.4 ± 4.1	−5.6	6/6
ML-GRF range (N)	57.4 ± 12.5	52.7 ± 13.8	−4.7	6/6
AP-COP range (mm)	111.4 ± 18.0	102.9 ± 18.9	−8.5	4/6
ML-COP range (mm)	33.6 ± 11.6	29.5 ± 11.6	−4.0	5/6
COP trajectory length (mm)	243.1 ± 63.6	211.0 ± 50.4	−32.1	5/6
COP rectangular sway area (mm^2^)	3,793.4 ± 1,485.5	3,103.0 ± 1,498.4	−690.5	6/6

### 3.5 Statistical comparison

Exploratory paired comparisons showed that several horizontal loading and center-of-pressure variables tended to decrease under the control condition. The Wilcoxon signed-rank test indicated significant reductions in the absolute peak of the anterior–posterior ground reaction force, the range of the anterior–posterior ground reaction force, the range of the medial–lateral ground reaction force, and the center-of-pressure rectangular sway area. The paired t-test also showed significant reductions in the range of the anterior–posterior ground reaction force, the medial–lateral center-of-pressure range, and the center-of-pressure rectangular sway area. The effect sizes for these variables were large, suggesting potentially meaningful biomechanical changes. The 95% confidence intervals for the mean paired differences were additionally reported to indicate the uncertainty of the estimated changes. Although several effect sizes were large, some confidence intervals were relatively wide because of the small sample size. In addition, because no formal correction for multiple comparisons was applied, the p-values should be interpreted cautiously and should not be considered confirmatory. The results of the exploratory paired statistical comparisons are shown in Table 3, and the mean paired differences with 95% confidence intervals are shown in Table 4.

**TABLE 3:** Exploratory paired statistical comparisons between the non-control and control conditions. Note: Statistical analyses were exploratory because of the small sample size. The results should be interpreted together with the consistency of change across participants, the confidence intervals shown in Table 4, and biomechanical relevance. GRF, ground reaction force; COP, center of pressure; AP, anterior–posterior; ML, medial–lateral.

Variable	Wilcoxon p-value	Paired t-test p-value	Effect size dz
Absolute peak AP-GRF	0.031	0.046	−1.08
AP-GRF range	0.031	0.030	−1.22
ML-GRF range	0.031	0.087	−0.87
ML-COP range	0.063	0.028	−1.25
COP trajectory length	0.094	0.082	−0.89
COP rectangular sway area	0.031	0.026	−1.28
Peak vertical GRF	1.000	0.542	−0.27
Vertical impulse	1.000	0.997	−0.00

**TABLE 4:** Mean paired differences and 95% confidence intervals between the non-control and control conditions. Note: Mean paired difference was calculated as the control condition minus the non-control condition. The 95% confidence interval indicates the confidence interval of the mean paired difference. Negative values indicate lower values under the control condition compared with the non-control condition. GRF, ground reaction force; COP, center of pressure; AP, anterior–posterior; ML, medial–lateral.

Variable	Mean paired difference	95% CI
Absolute peak AP-GRF (N)	−4.68	−9.24 to −0.12
AP-GRF range (N)	−5.59	−10.38 to −0.80
ML-GRF range (N)	−4.69	−10.37 to 0.99
ML-COP range (mm)	−4.01	−7.36 to −0.65
COP trajectory length (mm)	−32.06	−69.94 to 5.83
COP rectangular sway area (mm2)	−690.49	−1,257.10 to −123.87
Peak vertical GRF (N)	−4.28	−21.08 to 12.53
Vertical impulse (Ns)	−0.06	−37.77 to 37.66

### 3.6 Summary of main findings

Overall, the control condition reduced horizontal ground reaction force variability and center-of-pressure sway during stepping, whereas vertical ground reaction force peak and vertical impulse remained largely unchanged. In particular, the reductions in anterior–posterior ground reaction force range, medial–lateral ground reaction force range, and center-of-pressure sway area were consistently observed across all six participants. These findings suggest that the insole-type active assist device may improve loading stability during stepping by reducing horizontal force variability and center-of-pressure sway without substantially altering vertical loading.

## 4 Discussion

The present study investigated the effect of an insole-type active assist device on ground reaction force and center-of-pressure behavior during on-the-spot stepping in patients with medial knee OA. The main finding was that the control condition reduced horizontal ground reaction force variability and center-of-pressure sway compared with the non-control condition. In particular, the ranges of the anterior–posterior and medial–lateral ground reaction force components decreased in all six participants. Similarly, the center-of-pressure rectangular sway area also decreased in all participants. In contrast, peak vertical ground reaction force and vertical impulse showed little change between conditions. These findings suggest that the device may improve loading stability during stepping by reducing horizontal loading variability and center-of-pressure sway without substantially altering the magnitude of vertical loading.

The reduction in horizontal ground reaction force variability is an important finding because dynamic loading instability is closely related to the biomechanical characteristics of medial knee OA. Recent reviews have emphasized that biomechanical assessment remains central to understanding OA progression and evaluating loading modification strategies (Mündermann et al., 2024). Varus thrust and abnormal frontal-plane motion during the stance phase have been associated with medial knee OA progression and dynamic knee instability (Chang et al., 2004; Kuroyanagi et al., 2012). Although the present study did not directly calculate the knee adduction moment, the observed reduction in horizontal ground reaction force variability may reflect a more stable interaction between the lower limb and the ground during weight acceptance. Because the device provides heel-eversion assistance to guide the ankle toward a more neutral alignment, it may have reduced abrupt or excessive horizontal loading during stance. However, this proposed mechanism remains hypothetical. Because knee adduction moment, frontal-plane knee mechanics, and ankle kinematics were not directly measured in the present study, the relationship among ankle alignment correction, center-of-pressure behavior, horizontal ground reaction force variability, and medial knee loading requires confirmation in future studies. Therefore, the observed reductions in horizontal ground reaction force variability and center-of-pressure sway should not be interpreted as direct evidence of reduced medial compartment loading.

The present findings are also consistent with the biomechanical rationale of insole based interventions for medial knee OA. Previous studies have shown that lateral wedge insoles can modify center-of-pressure behavior and lower-limb loading in patients with medial knee OA (Hinman et al., 2009; Ferreira et al., 2022). In addition, lateral wedge insoles have been reported to alter lower-limb biomechanics, including ground reaction force and center-of-pressure behavior, in gait analysis studies (Du et al., 2024). Recent studies using wearable sensing insoles have also reported that lateral-wedge insoles can influence plantar-pressure patterns in patients with medial knee OA, supporting the importance of foot–ground interaction in this population (Hsu et al., 2023). However, conventional lateral wedge insoles provide static correction throughout the stance phase, regardless of step-to-step variations in foot and ankle posture. In contrast, the insole-type active assist device used in this study provides dynamic correction at heel contact. The reduction in horizontal ground reaction force range and center-of-pressure sway observed in the present study suggests that dynamic ankle alignment correction may offer a targeted approach for stabilizing loading behavior during stepping.

Center-of-pressure behavior provides useful information regarding postural steadiness and loading stability (Winter, 1995; Prieto et al., 1996). In the present study, center-of-pressure trajectory length and rectangular sway area decreased under the control condition. This indicates that the movement of the center of pressure during stance became smaller and more stable. Importantly, this change occurred without a substantial reduction in peak vertical ground reaction force or vertical impulse. Therefore, the reduction in center-of-pressure sway was unlikely to be explained simply by reduced loading or smaller stepping effort. Rather, the device may have altered the direction and stability of load transfer during stance.

These findings extend our previous feasibility study (Itami et al., 2026). The previous study demonstrated that the device could be safely used during an on-the-spot stepping task and that motion-capture-based evaluation could be successfully performed. However, that study was not designed to determine the biomechanical effectiveness of the device. The present secondary force-plate analysis provides additional preliminary evidence that the device may influence loading behavior during stepping. Specifically, the device did not markedly change vertical loading but reduced horizontal force variability and center-of-pressure sway. These findings may be consistent with improved loading stability; however, the underlying biomechanical mechanism remains hypothetical and should be verified in future studies using combined kinetic and kinematic analyses.

The clinical implication of this study is that dynamic ankle alignment correction may contribute to stabilizing lower-limb loading in patients with medial knee OA. Knee loading during daily activities is closely related to disease severity, pain, and functional limitations in people with knee OA (Wan et al., 2025). Although the present study used an on-the-spot stepping task rather than continuous walking or stair negotiation, reducing unstable horizontal loading during stance may be relevant for improving lower-limb loading control. The observed reductions in horizontal ground reaction force variability and center-of-pressure sway may therefore represent biomechanically relevant changes in loading stability during stepping. However, the clinical meaningfulness of the magnitude of these changes remains unclear. In particular, established thresholds for clinically meaningful reductions in horizontal ground reaction force variability or center-of-pressure sway during stepping in patients with medial knee OA are not currently available. Therefore, these findings should be interpreted as preliminary biomechanical findings rather than direct evidence of clinical benefit. Future studies should examine whether these force-plate changes are associated with clinically relevant outcomes, such as pain, perceived instability, physical function, walking ability, and medial knee loading.

Several limitations should be acknowledged. First, the sample size was small, and the analysis was exploratory. Although several variables showed consistent reductions across participants and large effect sizes, some confidence intervals were relatively wide, reflecting uncertainty in the estimated effects. In addition, multiple outcomes were examined without formal correction for multiple comparisons. Therefore, the statistical results should be interpreted as hypothesis-generating rather than confirmatory, and the results should not be interpreted as definitive evidence of clinical effectiveness. The present analysis also used range-based measures of horizontal ground reaction force, which may be sensitive to extreme values. Although incomplete stance phases were excluded and step-level values were averaged within each participant and condition, future studies should examine additional variability indices, such as standard deviation, coefficient of variation, impulse-based measures, and step-to-step variability, to provide a more comprehensive assessment of loading stability. Furthermore, detailed clinical and functional status measures, such as height, body mass, WOMAC, or KOOS scores, were not collected under the ethics-approved protocol of the original feasibility study. Therefore, the relationship between the observed force-plate changes and functional status could not be examined. Second, the task was limited to on-the-spot stepping. This task was selected for safety and prototype-related reasons, but it does not fully represent continuous walking or other daily activities. In addition, cadence and step timing were not externally controlled, and stance time showed a small reduction under the control condition. Although vertical loading measures were largely maintained between conditions, changes in stepping rhythm or stance duration may have influenced the horizontal ground reaction force and center-of-pressure outcomes. Future studies should control or standardize cadence and examine whether similar effects are observed during overground walking and other functional tasks. Third, knee adduction moment was not directly calculated, and medial compartment loading was not directly estimated. Therefore, the observed reductions in horizontal ground reaction force variability and center-of-pressure sway cannot be assumed to indicate reductions in medial compartment loading. In addition, the active device was applied to the right foot in all participants, and the right limb was used for the present force-plate analysis. Therefore, the analyzed limb did not necessarily correspond to the most symptomatic knee in every participant. However, all participants were confirmed by an orthopedic physician to have no severe bilateral deformity or significant difference in knee range of motion between the left and right knees. This should be considered when interpreting the biomechanical effects observed in the present study. Fourth, the timing of device activation was controlled by a physician-operated switch approximately 500 m before heel contact. Although the physician performed sufficient practice before data collection to standardize the switching procedure, the activation timing may still have varied across steps and participants. In addition, although the device was designed to guide the heel toward a neutral, approximately horizontal alignment at heel contact within a mechanical correction range of 0°–10°, actual ankle kinematics were not directly analyzed in the present force-plate study. Future systems should incorporate automatic sensor-driven control to trigger heel-eversion assistance more consistently, and future studies should combine force-plate analysis with ankle kinematics to verify whether the intended correction is achieved during each step.

Despite these limitations, the present study provides preliminary force-plate evidence that an insole-type active assist device can reduce horizontal ground reaction force variability and center-of-pressure sway during stepping in patients with medial knee OA. These findings support further investigation of dynamic ankle alignment correction as a biomechanical intervention for improving loading stability. Future studies with larger sample sizes, continuous gait tasks, and combined kinetic and kinematic analyses are needed to clarify the relationship among ankle alignment correction, center-of-pressure behavior, knee joint loading, and clinical outcomes.

## 5 Conclusion

This secondary force-plate analysis investigated the effects of an insole-type active assist device on ground reaction force and center-of-pressure behavior during on-the-spot stepping in patients with medial knee OA. The control condition reduced horizontal ground reaction force variability and center-of-pressure sway compared with the non-control condition, while peak vertical ground reaction force and vertical impulse remained largely unchanged. These findings suggest that dynamic ankle alignment correction may improve loading stability during stepping by reducing horizontal loading variability and center-of-pressure movement without substantially altering vertical loading.

Although the results provide preliminary biomechanical evidence supporting the potential usefulness of the device, they should be interpreted with caution because of the small sample size, exploratory secondary-analysis design, and the use of an on-the-spot stepping task. Future studies should include larger participant groups, continuous walking tasks, and combined kinetic and kinematic analyses. In particular, knee adduction moment, frontal-plane knee mechanics, ankle kinematics, and medial compartment loading should be evaluated to clarify whether the observed reductions in horizontal ground reaction force and center-of-pressure sway are associated with reduced medial knee loading and improved clinical outcomes in patients with medial knee OA.

## Data Availability

The data analyzed in this study is subject to the following licenses/restrictions: The data analyzed during the current secondary analysis are available from the corresponding author upon reasonable request. The data are not publicly available due to privacy and ethical restrictions. Requests to access these datasets should be directed to TI, itami@meiji.ac.jp.
